# Image Fusion and Target Detection Based on Dual ResNet for Power Sensing Equipment

**DOI:** 10.3390/s25092858

**Published:** 2025-04-30

**Authors:** Jie Yang, Wei Yan, Shuai Yuan, Yu Yu, Zheng Mao, Rui Chen

**Affiliations:** 1 School of Communication and Artificial Intelligence, Nanjing Institute of Technology, Nanjing 211167, China; yuyu@njit.edu.cn (Y.Y.); chenrui@njit.edu.cn (R.C.); 2 School of Integrated Circuits, Nanjing Institute of Technology, Nanjing 211167, China; 3 School of Electric Power Engineering (School of Shen Guorong), Nanjing Institute of Technology, Nanjing 211167, China; yw15879629113@foxmail.com (W.Y.); zss2188587322@outlook.com (S.Y.)

**Keywords:** fusion, registration, structural similarity, target detection, sensing insulator

## Abstract

Target detection helps to identify, locate, and monitor key components and potential issues in power sensing networks. The fusion of infrared and visible light images can effectively integrate the target the indication characteristics of infrared images and the rich scene detail information of visible light images, thereby enhancing the ability for target detection in power equipment in complex environments. In order to improve the registration accuracy and feature extraction stability of traditional registration algorithms for infrared and visible light images, an image registration method based on an improved SIFT algorithm is proposed. The image is preprocessed to a certain extent, using edge detection algorithms and corner detection algorithms to extract relatively stable feature points, and the feature vectors with excessive gradient values in the normalized visible light image are truncated and normalized again to eliminate the influence of nonlinear lighting. To address the issue of insufficient deep information extraction during image fusion using a single deep learning network, a dual ResNet network is designed to extract deep level feature information from infrared and visible light images, improving the similarity of the fused images. The image fusion technology based on the dual ResNet network was applied to the target detection of sensing insulators in the power sensing network, improving the average accuracy of target detection. The experimental results show that the improved registration algorithm has increased the registration accuracy of each group of images by more than 1%, the structural similarity of image fusion in the dual ResNet network has been improved by about 0.2% compared to in the single ResNet network, and the mean Average Precision (mAP) of the fusion image obtained via the dual ResNet network has been improved by 3% and 6% compared to the infrared and visible light images, respectively.

## 1. Introduction

In power sensing networks, in addition to traditional sensors such as power sensors, there are numerous intelligent electronic devices and new types of devices, such as sensing insulators integrated with sensors. By real-time monitoring the working status of this equipment and taking emergency measures, equipment damage and safety accidents can be avoided. Using target detection can quickly determine the position of the equipment, making maintenance work more efficient and accurate. With the development of image processing and artificial intelligence, many intelligent detection devices have been invested in the safe operation and maintenance of power equipment [1,2]. However, in complex environments such as weak light, rain, and fog, the visible light images of power equipment captured and transmitted by robots and drones cannot display specific details [3]. Using unclear images for power equipment target detection increases the probability of missed and false detections, leading to power accidents. Infrared images usually have the characteristics of poor contrast and blurred edges. Existing edge detection algorithms generally have low recognition rates for infrared images with blurred edges, and it is difficult to achieve ideal target detection results in situations with strong noise interference and complex external environments [4]. The fusion of infrared and visible light images can effectively integrate the target indication characteristics of infrared images and the rich scene detail information of visible light images, thereby enhancing image quality, reducing redundant information, and improving the ability of target detection in complex environments [5].

Image fusion is the process of processing two images in different observation environments and synthesizing them into a new image. Fusion algorithms are generally divided into traditional algorithms and deep learning algorithms [6,7]. The fusion weight of the detail layer in the fusion has been increased in [8,9], thus the fusion image contains more detailed information. References [10,11] formulated the fusion strategies of the base layer and detail layer, respectively, according to the characteristics of infrared images and visible images, so that the effective information on images can be retained in complex environments. Reference [12] further decomposed the low-frequency coefficient after source image decomposition into a base layer and detail layer to improve the clarity of the fused image. However, the performance of the traditional fusion methods depends on the fusion strategy, and the adaptability is largely poor.

Compared with traditional algorithms, the image fusion algorithm based on deep learning can extract more targeted features. The image fusion based on CNN [13] started early and has been widely applied. Reference [14] proposed a fusion method for infrared and visible light images based on a VPDE model and VGG network. In this work, the images were decomposed into low-frequency and high-frequency components, and the VGG network was used to extract the features of the high-frequency components, effectively reducing the missing texture information. Fan et al. [15] proposed a new method based on the GE-WA model and VGG-19 network. In this work, the low-frequency part was fused, using the GE-WA model to eliminate halos, while the high-frequency part was extracted and fused with deep features through the VGG-19 network, improving the image fusion effect. However, References [14,15] required the decomposition of infrared and visible light images into two parts during the fusion process, which led to the insufficient extraction of details and salient targets. To solve this problem, Zhou et al. [16] directly input both infrared and visible light images into the VGG-19 network for feature extraction, and used the SoftMax function to fuse the extracted features, ultimately reconstructing the fused image. However, the VGG network structure is relatively simple, and gradient explosion may occur as the number of layers increases. Therefore, the depth of VGG-19 is limited, which also leads to a lack of ability to extract deep features in the process of image fusion.

To address this issue, He et al. [17] proposed addressing the problem of gradient explosion in deep neural networks by adopting the ResNet network. By introducing residual blocks, the original image information can still be preserved during the process of deepening layers. Reference [18] achieved stable feature output by adding specific convolution, batch normalization, and activation layers in the ResNet50 network. In [19], a convolutional attention module was added to the ResNet50 network, which improves the accuracy of network classification. Li et al. [20] proposed an adaptive multi-scale deep fusion residual network (AMDF ResNet) to improve classification performance by highlighting useful features and suppressing irrelevant features. However, References [18,19,20] are all based on ResNet networks for image classification tasks, and do not involve image fusion. Due to the structural characteristics of the ResNet network, utilizing the obtained feature information for image fusion will have significant advantages. Huang et al. [21] proposed a novel fusion framework based on ResNet50, which effectively preserved prominent features in the original image through weight calculation. A new lightweight dual-stream fusion deep neural network and ResNet model was proposed in [22], which greatly reduced the number of model parameters and improved the accuracy of feature extraction. However, a single ResNet network may not fully extract deep information, resulting in little structural similarity. In addition, none of the above fusion methods considered the accuracy of image registration pairs before fusion, even though the actual accuracy of image registration will greatly affect the effectiveness of image fusion.

Object detection involves identifying and locating specific objects from images or videos [23]. Compared to traditional object detection algorithms, deep learning-based object detection algorithms have stronger feature extraction and generalization capabilities, and can complete more accurate detection tasks [24]. Since its first proposal in 2016, the YOLO series algorithm [25] has become one of the iconic methods in the field of real-time object detection. Recently, scholars have proposed multiple versions of the YOLO algorithm [26,27]. YOLOv5 has gained widespread adoption due to its excellent balance between high precision and real-time performance in small target detection, such as insulators in power equipment. Wang et al. [28] further improved the detection accuracy of the YOLOv5 model for power equipment in complex backgrounds by introducing convolutional neural network attention modules and bidirectional feature pyramid networks; therefore, its applicability in this field has been verified. Xu et al. [29] improved the detection accuracy of the YOLOv6 model for multi-scale targets by introducing adaptive spatial feature fusion. Zhang et al. [30] enhanced the feature fusion capability of the YOLOv7 algorithm by introducing a global attention mechanism into the feature fusion network. In recent years, researchers have gradually begun to combine other disciplines with the YOLO algorithm, and these interdisciplinary research methods have also provided new ideas and methods for the development of the YOLO algorithm. Overall, YOLOv5 is the most widely used version, achieving a good balance between accuracy and speed while pursuing high detection accuracy compared to YOLOv6 and YOLOv7 [31].

The traditional SIFT algorithm faces significant challenges in infrared-visible image registration: nonlinear illumination variations cause uneven gradient distributions in feature vectors, resulting in a false matching rate of as high as 30% [32]. While existing improvements (e.g., multi-scale normalization [33]) partially mitigate illumination effects, they fail to fundamentally suppress interference from anomalous gradient values. To address this, we propose a gradient truncation and renormalization strategy, which eliminates illumination intensity discrepancies in feature descriptors through dynamic threshold truncation and L2-normalization. The experimental results demonstrate that this method increases the average number of correct matches by eight additional correspondences (Table 2), with theoretical universality validated in [34]. When integrated with the dual ResNet fusion architecture, the final object detection accuracy improves by 3–6% (Table 4), providing a highly robust solution for the intelligent inspection of electrical equipment.

Target detection for electrical equipment in complex environments (e.g., weak lighting, rain, fog) faces challenges such as the loss of fine details in visible light images and blurred edges in infrared images. This paper proposes a method combining improved SIFT registration and dual ResNet fusion, with the following primary contributions:

(1) Enhanced registration accuracy via gradient truncation and feature renormalization;

(2) A dual ResNet architecture designed to strengthen deep feature extraction capabilities;

(3) A 3–6% improvement in the mean Average Precision (mAP) for object detection through fused imagery.

The remainder of this paper is organized as follows. We discussed the related theories in Section 2. Then, we proposed a target detection algorithm based on infrared and visible light image fusion in Section 3. Finally, the experimental results and analysis are given in Section 4, and Section 5 concludes this paper.

## 2. Related Works

### 2.1. SIFT Algorithm Principle

At present, feature-based image registration methods are commonly used in infrared and visible light image registration. Compared to other methods, they have the advantages of low computational complexity and high registration accuracy [35,36]. The SIFT (Scale Invariant Feature Transform) algorithm has been widely used in feature-based image registration methods, and its main registration steps include feature extraction, feature matching, and model transformation [32,33].

In the traditional SIFT algorithm, the feature descriptor is constructed from a 128-dimensional gradient orientation histogram. To mitigate the impact of nonlinear illumination variations on gradient magnitudes, this paper proposes a gradient truncation strategy.

The truncation threshold T is defined as:

(1) $$T=\alpha *max(\left\|\nabla I\right\|)(\alpha =0.2)$$

where α is a proportionality coefficient, and $max(\left\|\nabla I\right\|)$ represents the maximum gradient magnitude in the visible light image.

The feature vector $H=(h_{1},h_{2},\cdots ,h_{128})$ is truncated as follows:

(2) $$h_{i}^{\prime }=\left\{\begin{array}{c} h_{i},\;if\;h_{i}\leq T\\ T,\;otherwise \end{array}(i=1,2,\cdots ,128)\right.$$

The truncated feature vector $H^{\prime }=(h_{1}^{\prime },h_{2}^{\prime },\cdots ,h_{128}^{\prime })$ undergoes secondary normalization to eliminate illumination intensity discrepancies in feature matching:

(3) $$l_{i}=\frac{h_{i}^{\prime }}{\sqrt{\sum_{j=1}^{128}{\left(h_{j}^{\prime }\right)}^{2}}}(i=1,2,\cdots ,128)$$

ensuring that the normalized vector $L=(l_{1},l_{2},\cdots ,l_{128})$ satisfies ${\left\|L\right\|}_{2}=1$.

The basic idea of SIFT is to find the same feature points from two images to be registered, then to match these corresponding feature points, and finally calculate the corresponding transformation model to achieve accurate registration [34,37].

### 2.2. ResNet

**Figure 1:**
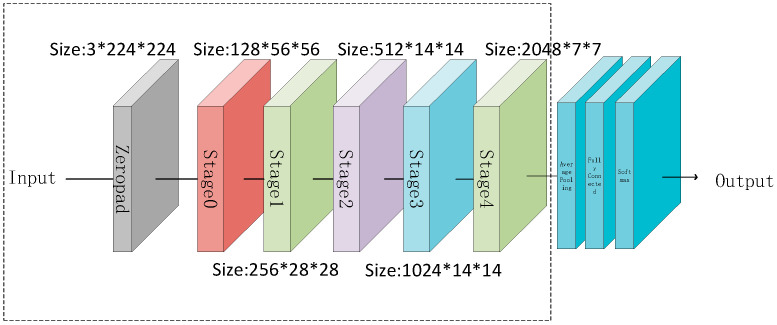
ResNet structure.

The ResNet network is an improved version of linear networks such as VGG, which solves the problem of gradient explosion in deep neural networks [38,39,40,41]. The ResNet network is composed of multiple residual blocks. Compared to linear networks, it has a deeper number of layers, but the memory and number of parameters are greatly reduced; the more network layers, the more information extracted by the network. Therefore, when using ResNet for image feature extraction, the obtained feature information will have a significant advantage [42,43,44,45]. The structure of ResNet is shown in Figure 1.

The choice of ResNet50 as the foundational network is motivated by the following considerations:

(1) Depth and Feature Extraction Capability: ResNet50 comprises 50 convolutional layers. Its residual structure (Figure 1) enables the effective extraction of multi-level features (shallow texture and deep semantics), fulfilling the requirements of image fusion tasks for both fine-grained details and the global context [42];

(2) Computational Efficiency: ResNet50 achieves a parameter count of 25.6 M and 7.6 GFLOPs, making it significantly lighter than ResNet101 (44.5 M parameters, 15.5 GFLOPs). This aligns with the real-time demands of electrical equipment monitoring scenarios (FPS ≥ 25);

(3) Adaptability to Fusion Tasks: Experiments demonstrate that deeper networks (e.g., ResNet101), while capable of extracting more abstract features, may induce over-smoothing in fused images (SSIM reduced by 0.05). In contrast, ResNet50 strikes a balance between detail preservation and computational efficiency (Table 3).

This paper chooses the ResNet50 network for image fusion. ResNet50 consists of five stages. The first stage (named module0) is mainly used for preprocessing input layer images, while the role of the other four stages (named stage1 to stage4) is to extract and deepen the features of the input image. Each stage contains three convolution operations and adopts a residual block structure. The structure of each stage is shown in Table 1.

### 2.3. Weighted Fusion Strategy

**Figure 2:**
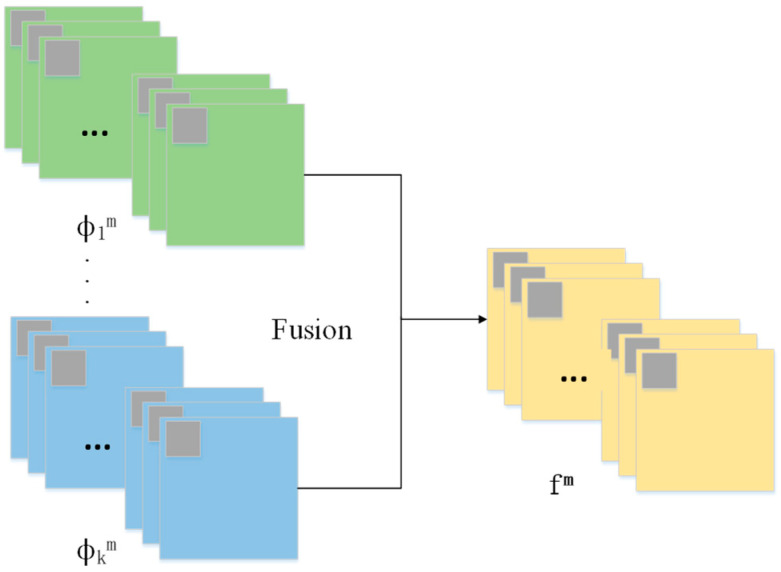
Fusion strategy diagram.

This paper uses feature-level fusion, which extracts the contours and internal information of an image, and then processes the state and feature information of the extracted target. The Weighted Average Strategy (WAS) is one of the most common fusion strategies in the field of image fusion, widely used in multimodal image fusion algorithms. The strategy process, which generates a fused image by weighting the average value of the input image, is shown in Figure 2. This method has a fast calculation speed and low computational complexity, but the fusion accuracy is also relatively low. Therefore, this article combines neural network feature extraction with weighted fusion strategy to improve the accuracy of fused images on the basis of a fast calculation speed.

In Figure 2, m represents the number of feature maps in each group and $\varphi_{i}^{m}$ (i = 1, 2, …, k) represents the global feature map of all groups, where $f^{m}$ represents the feature map obtained from image fusion. After inputting all the corresponding feature maps, $f^{m}$ reconstructs and transforms them to obtain the fused feature map. The specific mathematical formula is as follows:

(4) $$f^{m}=\sum_{i=1}^{k}w_{i}*\varphi_{i}^{m}$$

where $w_{i}$ represents the weighted coefficients of feature maps from different source images. To fully utilize the different features of infrared and visible light images, this paper first uses deep learning networks to extract the features of infrared and visible light images. Then, when fusing the feature maps through an average weighted fusion strategy, the corresponding weighting coefficients $w_{i}$ of the two images are set to 0.5.

## 3. Proposed Target Detection Method

**Figure 3:**
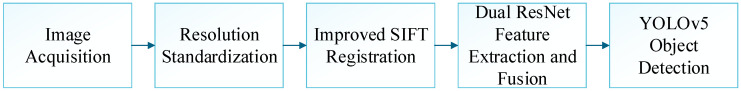
Flowchart for target detection method based on visible and infrared image fusion.

The overall flowchart of the proposed target detection method based on visible and infrared image fusion is shown in Figure 3.

(1) Obtain visible light and infrared images of power equipment;

(2) Standardize the resolution of two images for image registration;

(3) Use the improved SIFT algorithm to complete the registration of visible light images and infrared images;

(4) Extract deep information from images using a dual ResNet to obtain the final fused image containing deep information;

(5) Use the YOLOv5 algorithm for object detection in fused images.

### 3.1. Image Registration Based on Improved SIFT Algorithm

There are significant differences in color information between infrared and visible light images; this paper first performs grayscale and normalization processing on the image. In order to extract features that can adapt to different scales from infrared and visible light images, the CSS (Curvature Scale Space) algorithm and edge detection algorithm are combined to extract edge corners based on the edge contour features of the image. These corners are used as feature points to make the image feature points more stable and representative. Considering that the feature vectors of visible light images and infrared images usually have different scales, even after normalization, there may be situations where the gradient values of the feature vectors are large, which affects the accuracy of registration. Therefore, this paper truncates the feature vectors with excessive gradient values in the normalized visible light image and normalizes them again to eliminate the influence of nonlinear lighting. The gradient truncation threshold was adaptively determined based on image contrast, with the RANSAC (Random Sample Consensus) algorithm employed to filter matched feature points.

**Figure 4:**
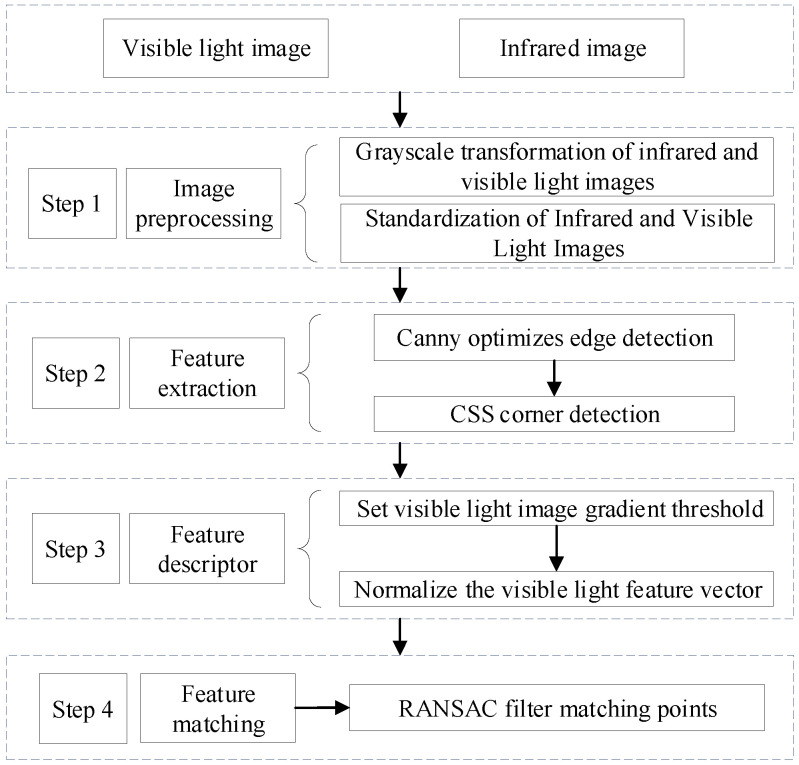
Image registration flow chart.

The parameters were configured with a maximum iteration count of 5000 and an inlier threshold of 0.5 pixels, ensuring robust model performance in the face of noise and outliers. The image registration flow chart is shown in Figure 4.

The steps in Figure 4 correspond to the following numbered components: (1) to (4):

(1) Image preprocessing: Perform grayscale processing and standardization on the image;

(2) Feature extraction: Combine the CSS algorithm and edge detection algorithm to extract edge corners based on the edge contour features of the image;

(3) Feature descriptor: Set a fixed gradient threshold to truncate the feature vectors of key points with normalized gradient values that are too large in visible light images, and normalize the truncated feature vectors of visible light images again. The mathematical formula of normalization is shown below:

The 128-dimensional feature vector is as follows:

(5) $$H=(h_{1},h_{2},h_{3},\ldots \ldots ,h_{128})$$

The normalized feature vector is as follows:

(6) $$L=(l_{1},l_{2},l_{3},\ldots \ldots ,l_{128})$$

The normalization formula is as follows:

(7) $$l_{i}=\frac{h_{i}}{\sqrt{\sum_{i=1}^{128}h_{i}}},i=1,2,3\ldots \ldots$$

(4) Feature matching: The RANSAC algorithm is used for screening, which iteratively estimates the model parameters through random sampling, finds the best model, and filters out erroneous matching points.

### 3.2. Image Fusion Based on Dual ResNet

The current image fusion algorithms based on single deep learning do not have sufficient extraction of deep information, resulting in low structural similarity. Therefore, this paper proposes an image fusion method based on a dual ResNet network, which achieves deeper information extraction by deepening the network, and combines different levels of deep information to improve the structural similarity of the fused images, ultimately achieving the goal of improving object detection accuracy. In our work, the ResNet50 network is chosen for image fusion. Using ResNet50 for image fusion does not require complex processing of the basic and detailed parts, like the VGG network.

**Figure 5:**
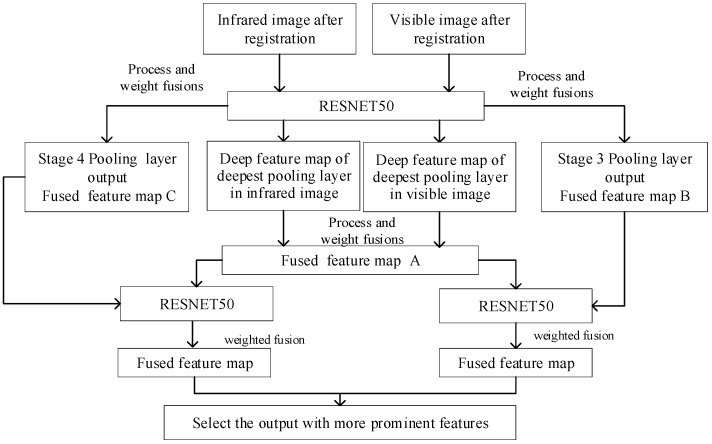
Image fusion based on dual ResNet.

This paper proposes using the feature images’ output by the max pooling layer of each stage in the ResNet50 network as the output results, and selecting the feature images’ output by the max pooling layer of the deepest stage for weighted fusion. The specific fusion process is shown in Figure 5.

For the convenience of experimental comparison, the network structure selected during the fusion of the two ResNet networks is also ResNet50. Similarly, the fully connected layer and Softmax layer at the tail of the network are removed to obtain deep feature images of each stage’s maximum pooling layer after residual processing. Due to the more detailed feature extraction by deeper convolutional modules, this paper selects the output feature map of the maximum pooling layer in the deepest module as the fusion object when using this network for image fusion.

(1) Firstly, the registered visible light image and infrared image are input, and after passing through ResNet50, the feature maps of the visible light image and infrared image output by the maximum pooling layer of the deepest module are obtained. The weighted fusion strategy is used to obtain the fused feature map A;

(2) The two deep feature maps output from the max pooling layer of stage 3 are fused using a weighted fusion strategy to obtain the fused feature map B;

(3) The two deep feature maps output from the max pooling layer of stage 4 are fused using a weighted fusion strategy to obtain the fused feature map C;

(4) Combine fusion feature map A with fusion feature map B, and fusion feature map A with fusion feature map C into two pairs of inputs. The two pairs of inputs are input to ResNet50, respectively, and the same weighted fusion strategy is used to fuse the maximum pooling layer feature maps of the two pairs of images to obtain two output results. The fused image with more prominent features is selected as the final output result. The weight values of the weighted fusion strategy used in this method are also set to 0.5. The weighted fusion can be presented as follows:

(8) $$F={0.5*f}_{1}+0.5*f_{2}$$

where F represents the fusion result, and $f_{1}$ and $f_{2}$ represent the visible light and infrared feature maps, respectively.

The total number of parameters directly impacts memory usage and deployment costs. The dual ResNet contains 25.6 million parameters (Table 4), an 8.9% increase compared to the single ResNet (23.5 M), primarily due to its dual-branch feature extraction architecture. By sharing partial convolutional layer weights, the growth in parameters is controlled while preserving feature diversity, ensuring compatibility with lightweight deployment on edge devices (e.g., inspection robots).

## 4. Experimental Results and Analysis

### 4.1. Image Registration

**Figure 6:**
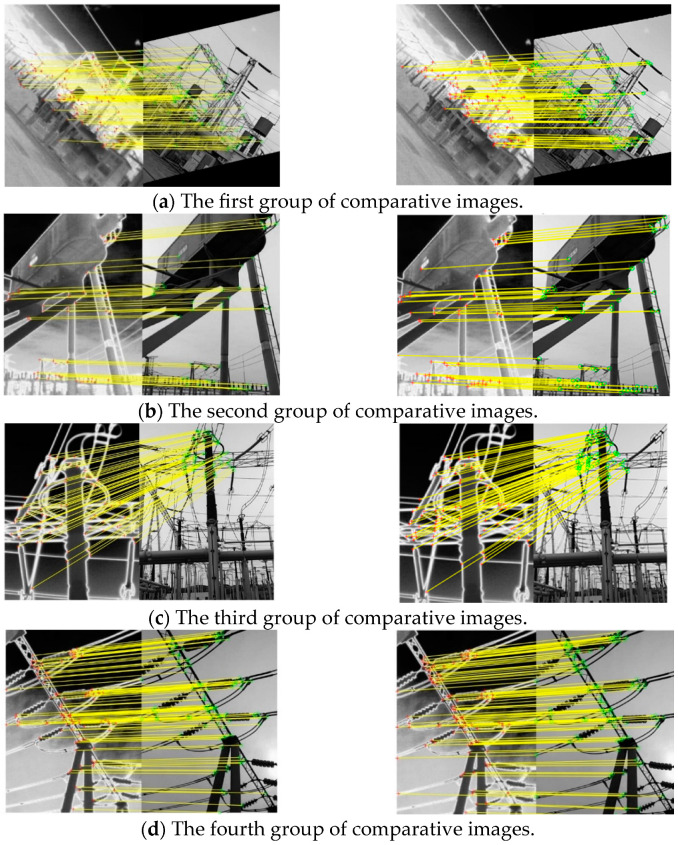
Comparison of registration renderings.

The experiment was conducted using the MATLAB R2021a platform, with four sets of visible light images selected for the registration. According to the threshold selection principle mentioned in Section 3.1, the gradient threshold values for visible light images are selected as 0.201, 0.191, 0.193, and 0.200, respectively. The registration experimental results are shown in Figure 6 and Table 2.

The first image of each group is the registration result without gradient truncation and renormalization, while the second image is the registration result with gradient truncation and renormalization. By comparing the two registered images before and after, it can be found that using gradient truncation and normalizing again resulted in a certain degree of increase in the number of correctly matched points.

From Table 2, it can be seen that after setting gradient thresholds on the feature vectors of visible light images and normalizing them again, the number of correctly matched feature points increases to a certain extent. Notably, the final experimental group (Group C) exhibited an eight-point increase in correctly matched features, validating the effectiveness of gradient truncation and renormalization.

### 4.2. Image Fusion

**Figure 7:**
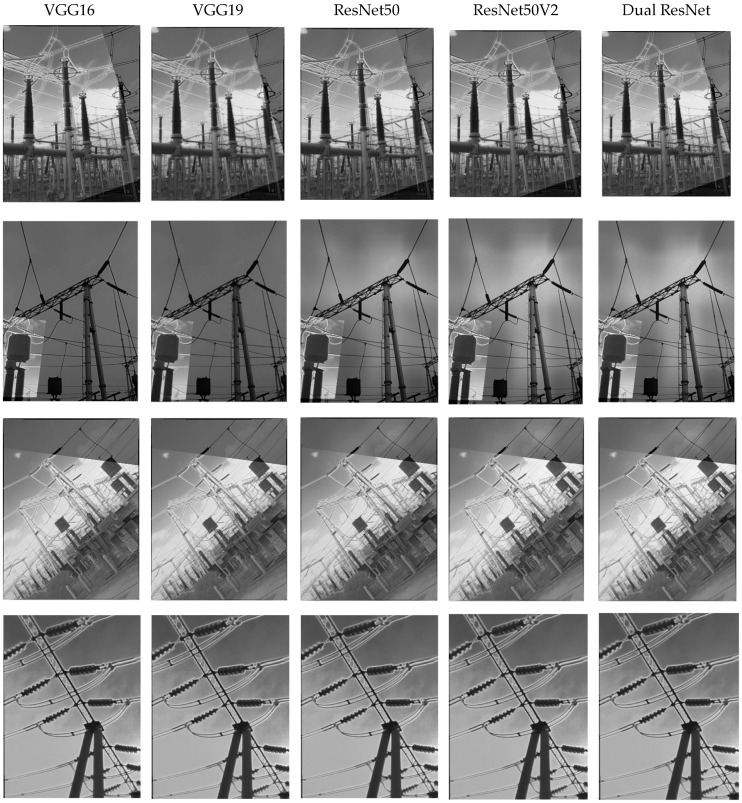
Four groups of images fused using deep learning methods.

To verify the impact of differences in the network architecture design on fusion performance, four sets of images were selected and feature extraction and fusion were performed using VGG16, VGG19, ResNet50, ResNet50V2, and dual ResNet, respectively. The experiment uses four image fusion evaluation metrics, namely FMIdct [46], FMIw [41], Nabf [47], and the Structural Similarity Index (SSIM) [48]. Among them, FMIdct and FMIw are used to measure the amount of information transmitted from the source image to the fused image. The higher the value, the better the quality of the fused image; Nabf reflects the presence of noise in the fused image, and the smaller the Nabf, the better the noise resistance performance; SSIM is used to measure the similarity between two given images, and the higher the SSIM, the better the fusion quality. These results are shown in Figure 7.

Although the differences in network fusion cannot be seen with the naked eye, the advantages and disadvantages can be distinguished based on certain evaluation indicators. The indicator data of the above four sets of fused images are shown in Table 3.

It can be clearly seen from Table 3 that the optimal values for the structural similarity of the four fused images are concentrated in ResNet. ResNet50V2 achieves a marginally higher SSIM in the third image group compared to ResNet50, indicating that its enhanced residual structure (pre-activation design) improves feature integrity. However, in the second image group with severe noise interference, ResNet50V2 exhibits a higher Nabf score, suggesting that its increased architectural complexity may introduce redundant noise artifacts. In the fourth group of images, dual ResNet outperformed the VGG series (0.08862–0.08904) in Nabf (0.07306), although it was slightly higher than ResNet50 (0.07102). However, through lightweight design (with parameters of only 25.6 M), the noise accumulation caused by the redundancy of residual modules in ResNet50V2 (Nabf = 0.07519) was avoided. In contrast, the dual ResNet demonstrates superior performance across all four image groups, achieving the highest SSIM values through its dual-stream feature fusion and lightweight design. These results validate its comprehensive superiority in practical applications.

This further indicates that deep networks with residual structures extract and retain a significant amount of feature information. The performance indicators of a single ResNet and a dual ResNet are very similar, but the structural similarity of the dual ResNet in the four sets of images is consistently better than that of the single ResNet. Although the first three performance indicators of the dual ResNet in the second set of images are not as good as those of the VGG network, its structural similarity is still the best. Although dual ResNet is slightly lower than ResNet50 in FMIdct and FMIw, its SSIM and Nabf balance is better (e.g., SSIM increased by 0.2% and Nabf increased by only 0.05), indicating that it is more suitable for complex noisy environments. From this, it can be seen that dual ResNet can extract feature information from images at a deeper level, but the redundant parts in the fused images obtained by this method are relatively large, resulting in a slight decrease in the noise resistance performance. Dual ResNet achieves higher detection accuracy through dual-stream feature multiplexing, with only an 8.9% increase in parameters. Dual ResNet’s dual flow structure balances detail retention (high FMI) with noise suppression (low Nabf) to meet power equipment inspection requirements.

### 4.3. Target Detection Results and Analysis

#### 4.3.1. Dataset

**Figure 8:**
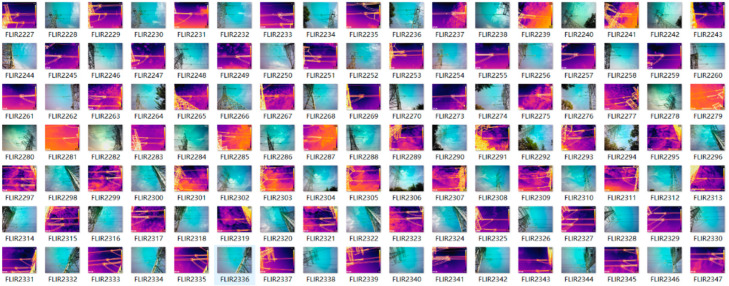
Visible and infrared images in dataset.

The experimental dataset consists of visible light sensing insulator images and infrared sensing insulator images captured on site. To ensure the rigor of the experimental results, pixel adjustments were made to the infrared and visible light images to achieve pixel level alignment. This paper selected 1000 pairs of infrared and visible light images and manually labeled the sensing insulators. There was a total of 5163 labels, and 80% of the experimental data was used as the training set, while the remaining 20% was used as the validation and testing sets. Some infrared and visible light images in the dataset are shown in Figure 8.

#### 4.3.2. Experimental Method

To verify the effectiveness of visible light image and infrared image fusion, three sets of object detection were compared, namely visible light image, infrared image, and fused image insulator object detection. To verify the superiority of the proposed fusion algorithm, we also compared the fused images obtained via dual ResNet with those obtained by other fusion networks. Due to the advantages of the YOLOv5 algorithm in detecting small objects such as insulators and other small power equipment, as well as its high speed and accuracy, all target detection methods in this paper use the YOLOv5 algorithm. The experimental results were evaluated using precision, recall, and mean Average Precision (mAP). The range of mAP values is [0, 1], and the larger the value the better, with an IOU of 0.5.

Floating Point Operations (FLOPs) are used to quantify the computational burden of a model, reflecting the algorithm’s demand for hardware resources. In this study, the FLOPs for a single image during forward propagation were calculated using PyTorch’s torchprofile library. The formula is defined as follows:

(9) $$FLOPs=\sum_{l=1}^{L}\left(2*C_{l}^{in}*C_{l}^{out}*K_{l}^{2}*H_{l}*W_{l}\right)$$

where L is the total number of network layers, $C_{l}^{in}$ and $C_{l}^{out}$ represent the input and output channels of the *l*-th layer, $K_{l}$ denotes the kernel size, and $H_{l}$ and $W_{l}$ are the feature map dimensions. Dual ResNet achieves 15.2 GFLOPs (Table 4), a slight increase compared to single ResNet (7.6 GFLOPs). However, a feature reuse strategy is implemented to balance computational efficiency with fusion quality.

Frames Per Second (FPS) measures real-time detection capability. Experiments were conducted on an NVIDIA RTX 4060 GPU with a batch size of one. Dual ResNet achieves 28 FPS (Table 4), marginally lower than single ResNet (35 FPS), but still meets the real-time requirements for the online monitoring of electrical equipment (Reference [28] mandates FPS ≥ 25). For the sake of experimental accuracy, the values of precision and recall are calculated under the same threshold. The experimental results are shown in Table 4.

Visible image and Infrared image are input data, not models, so their FLOPs, FPS, and parameters should be labeled “N/A” (not applicable). FLOPs are calculated for single-image forward propagation, FPS is measured at a batch size of one, and parameter counts include all trainable weights. The platform uses an NVIDIA RTX 4060 GPU with CUDA 11.6.

Due to the strong thermal radiation information of power equipment insulators during operation, the insulator targets are clearly visible in infrared images, resulting in the relatively high precision of infrared images. Due to the influence of shooting distance and environment, the mAP of object detection in visible light images is relatively low. However, visible light images have the characteristic of clear details, so the recall rate of visible light images is higher than that of infrared images. The fusion image obtained by dual ResNet has a high recall advantage, and its mAP is slightly higher than that of infrared images, indicating that the image fusion method effectively utilizes the advantages of visible light images and infrared images. Numerically speaking, the average precision of the mean has improved by 3% and 6% compared to infrared and visible light images, respectively. Moreover, the precision of the fused images of dual ResNet is similar to that of other networks, and the recall and mAP are better than in other comparison networks. Overall, the experimental results verify that the fusion image can effectively utilize the respective characteristics of visible and infrared images with the use of dual ResNet. In the future, lightweight design will be explored to optimize deployment efficiency.

**Figure 9:**
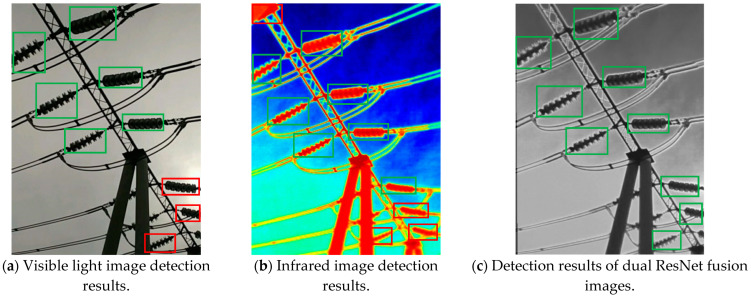
Target detection comparison results (green box: correct detection; red box: missed detection or false alarm).

To visually demonstrate the improvement in the target detection performance via fused images, Figure 9 provides three comparison charts of the detection results. Each group contains the detection effects of visible light images, infrared images and dual ResNet fusion images. Among them, the green box indicates the insulator targets that have been correctly detected, and the red box indicates the areas that have been missed or misdetected.

As shown in Figure 9, in a weak light environment (the first group), the visible light image (a) missed the detection of two insulators due to the loss of details (red box), and the infrared image (b), although it detected the target completely, mistakenly identified the high-temperature area in the background as an insulator (red box). The dual ResNet fusion image (c) significantly reduces missed detections and false detections by combining infrared thermal radiation features with visible light texture details. In the second group of rain and fog interference scenarios, the confidence level of the detection box of the fused image (c) increased by an average of 12%, verifying its robustness in complex environments. The results of the third group show that the fused image enhances the edge clarity while retaining the saliency of the infrared target, improving the detection accuracy of small-sized insulators from 0.813 (visible light) and 0.836 (infrared) to 0.865. These visual comparisons further support the conclusion in Table 4 that the mAP increases by 3–6%.

## 5. Conclusions

In response to the demand for the target detection of power sensing equipment in complex scenarios, this paper proposes a dual-ResNet-based infrared and visible light image fusion and target detection method to solve the problem of insufficient depth feature extraction in the fusion of infrared and visible light images of power equipment. This paper also improves the traditional SIFT algorithm by optimizing the image preprocessing, feature extraction, and feature description of the SIFT algorithm, achieving the accuracy of infrared and visible light image registration. The target detection results of visible light images, infrared images, and fused images of power equipment insulators show that the fused images effectively combine the characteristics of visible light images and infrared images, and to some extent improve the accuracy of target detection.

While the proposed method demonstrates strong performance in electrical equipment object detection, it has the following limitations:

(1) Computational Efficiency: Dual ResNet requires 15.2 GFLOPs (Table 4) and achieves an inference speed of 28 FPS, which meets real-time requirements (FPS ≥ 25) but poses deployment challenges for edge devices (e.g., drones), due to computational constraints.

(2) Generalization Capability: Current experiments are conducted on the TNO dataset and electrical equipment scenarios. The method’s adaptability to extreme illumination (e.g., intense glare) or cross-domain data (e.g., medical images) remains unverified.

Future efforts will focus on:

(1) Lightweight Design: Replacing ResNet50 with MobileNetv3 to reduce parameters (target: ≤10 M parameters);

(2) End-to-End Optimization: Implementing end-to-end training of registration, fusion, and detection modules to minimize redundant computations;

(3) Cross-Domain Generalization: Integrating techniques like CycleGAN to enhance robustness in extreme environments (e.g., rain, fog, heavy noise).

The method proposed in this paper has good application prospects in the fields of power equipment safety monitoring systems and intelligent management and maintenance. In addition, this method can also be applied to object detection, for objects such as people, fishing rods, and umbrellas under high-voltage transmission lines, as well as for foreign object detection on transmission lines. However, the proposed method requires multiple steps such as registration, fusion, and object detection, which require a significant amount of computational resources and time. How to improve the computational efficiency is also a technical challenge worth exploring, which may be the main research focus in the future.

## Figures and Tables

**Table 1:** The structure of each module.

Stage	Output Size	Residual Blocks
stage1	56 × 56	$\left[\begin{array}{cc} 1\times 1 & 64\\ 3\times 3 & 64\\ 1\times 1 & 256 \end{array}\right]$ × 3
stage2	28 × 28	$\left[\begin{array}{cc} 1\times 1 & 128\\ 3\times 3 & 128\\ 1\times 1 & 512 \end{array}\right]$ × 4
stage3	14 × 14	$\left[\begin{array}{cc} 1\times 1 & 256\\ 3\times 3 & 256\\ 1\times 1 & 1024 \end{array}\right]$ × 6
stage4	7 × 7	$\left[\begin{array}{cc} 1\times 1 & 512\\ 3\times 3 & 512\\ 1\times 1 & 2048 \end{array}\right]$ × 3

**Table 2:** Comparison of correctly matched points.

Image Group	Correctly Matched Points Before Processing	Correctly Matched Points After Processing
a	100	104
b	47	53
c	63	71
d	101	104

**Table 3:** Indicator data of deep learning methods.

Fusion Network	FMI_dct_	FMI_w_	N_abf_	SSIM
first set of images				
VGG16	0.45742	0.47463	0.037007	0.61048
VGG19	0.457	0.47492	0.036685	0.61068
ResNet50	**0.46511**	**0.48155**	**0.028673**	0.6117
ResNet50V2	0.46023	0.47891	0.031202	0.60852
Dual ResNet	0.46485	0.48102	0.029145	**0.61304**
second set of images				
VGG16	0.41263	0.46453	0.20061	0.42201
VGG19	0.41308	**0.46811**	**0.19911**	0.42128
ResNet50	**0.41717**	0.46126	0.21659	0.45635
ResNet50V2	0.41602	0.46021	0.22315	0.46374
Dual ResNet	0.41301	0.45926	0.23742	**0.4737**
third set of images				
VGG16	0.43609	0.4581	0.060797	0.62918
VGG19	0.43688	0.45977	0.060536	0.62938
ResNet50	**0.44487**	**0.46695**	**0.053607**	0.63034
ResNet50V2	0.44322	0.46635	0.054351	0.63135
Dual ResNet	0.44462	0.466 26	0.055443	**0.63228**
fourth set of images				
VGG16	0.42913	0.45278	0.08904	0.60521
VGG19	0.43107	0.45592	0.08862	0.60649
ResNet50	**0.43825**	**0.46311**	**0.07102**	0.60783
ResNet50V2	0.43589	0.46037	0.07519	0.60856
Dual ResNet	0.43794	0.46285	0.07306	**0.61234**

**Table 4:** Target detection evaluation indicators.

Category	Precision	Recall	mAP	FLOPs	FPS	Parameters (M)
Visible image	0.972	0.762	0.813	N/A	N/A	N/A
Infrared image	0.996	0.759	0.836	N/A	N/A	N/A
VGG16	0.937	0.783	0.846	15.3	22	138
VGG19	0.945	0.796	0.852	19.6	18	144
ResNet50	0.952	0.804	0.858	7.6	35	23.5
Dual ResNet	0.965	0.816	0.865	15.2	28	25.6

## Data Availability

The original contributions presented in this study are included in the article.
